# To Correspondents

**Published:** 1858-04

**Authors:** 


We have received for publication the following original articles, which will appear as space will allow, viz: Traumatic

Occlusion of the Vagina, by Ernest Schmidt, M.D., Chicago, Ill.; Milk-Sickness, by J. P. DeBruler, M.D., Rockport, Ind.; Surgical Cases, by J. W. Benson, M.D., Chicago, Ill.; Cases of Ovarian Dropsy, by J. H. Brower, M.D., Lawrenceburgh, Ind.; Turpentine in Hæmoptysis, by Jas. M. Kindall, Wabash, Ind.; Malformation, by S. W. Wallace, M.D., Black Earth, Wis.; A Case in Practice, by L. H. Angell, M.D., of Aurora, Ill. We have also on our table a communication, with post mark Rochester, Ind., but unfortunately we cannot determine its paternity, as its author forgot to append his name to it. It is a good paper, and will be published if the writer will furnish us with his name. And here we hope to be pardoned for chiding our medical friends generally for not communicating their experience to the profession through the medical journals of the day. Thousands of valuable facts,—facts that would come opportunely to the rescue of perplexed brethren in their efforts to save life—are lost through the false and *inexcusable* modesty or laziness of men of vast experience and profound learning, in not recording and disseminating their knowledge in this way. Many things that to them seem commonplace, from their familiarity, are known to no one else perhaps, and the knowledge pertaining to them dies—when they die. This is a sin against common sense and professional liberality that we are surprised to see so generally prevailing in our ranks. We are glad to know and be able to inform our readers that there is hope of reform in this matter, particularly among our young men. Old sinners may not and perhaps never will repent, and what is worse, true to their obdurate and tenacious habits of detraction, they are ever ready to sneer at any, even the slightest awkwardness of their juniors in their laudable efforts to form better habits for themselves. To these old aristocratic, silent gentlemen we have nothing more to say than, Young America like, "keep your silence" before your superiors,—and the first one who writes even scoldingly for the *Journal* we will readily and willingly relieve from that interdiction—but the young men in the great and glorious field of scientific research, we would advise to be careful what habits they allow themselves to fall into. Resolve, and begin to put that resolution into execution at once, that you will be active, working, liberal coadjutors of your most worthy compeers of the profession; that you will not be mere machines, upon which the accumulating flood of knowledge acts, only to grind out dimes. Write! write! And if you do not begin early, you will never begin at all: just allow the first decade of your professional life to pass in unbroken silence, and you are dumb for all future time. This function is not an exception to the general physiological rule, that inaction incapacitates. We hope you will begin in earnest, and now, and every experienced writer will forgive your first efforts if they should not be finished ones. They remember their own too well not to be lenient in this respect, and they are not half so exacting as those old fogies who have emasculated themselves by withholding their progeny until they are incapable of a respectable procreative effort. Do not be afraid of any such eunuchs in the profession, and take warning by their example. The first effort is almost sure to decide the matter; begin, and you will continue; make one step in the right direction, and you will progress easily. You cannot expect to satisfy your wishes in your first trial. Of course, your paper will not be as good as you will write after much exercise and experience, but it will show promise of a determination, that must work good results. Every time you produce an article, you will increase in strength, and what is still better, have a more correct appreciation of your powers. You will soon find that editors and authors all must wear literary swaddling, before their legs are long enough to stride in breeches among giants of learning, and that you are not a pigmy because your infantile extremities are not developed to the size of a man. Our word for it, every time you produce a good article for the *Journal*, you will be several inches taller, not merely in your own estimation, but in the good opinion of all your readers; and at the end of a long life spent in useful, liberal, and scientific converse with your brethren in this and every other available way, you will have the consolation to know that you have made every reasonable effort to do your duty toward men, to whom you are indebted for everything you have and are. We would be glad to write more upon this interesting—nay, to young medical men, *all-important* subject, but the printer interrupts us with the not generally annoying announcement, "enough copy, sir!" B.

